# EEG focal delta slowing in focal epilepsy – A didactic review

**DOI:** 10.1016/j.cnp.2025.09.001

**Published:** 2025-09-11

**Authors:** Laurent Sheybani, Pia De Stefano, Margitta Seeck, Serge Vulliémoz, Pierre Mégevand

**Affiliations:** a Department of Clinical and Experimental Epilepsy, University College London, London, United Kingdom; b Department of Clinical Neuroscience, Geneva University Hospitals and University of Geneva, Geneva, Switzerland; c Neuro-Intensive Care Unit, Department of Intensive Care, University Hospital of Geneva, Geneva, Switzerland; d Center for Biomedical Imaging (CIBM) Lausanne-Geneva, Geneva, Switzerland; e Department of Fundamental Neuroscience, University of Geneva Faculty of Medicine, Geneva, Switzerland

**Keywords:** Epilepsy, Focal slowing, Seizure, Lesional epilepsy, Prognosis

## Abstract

Focal slowing in scalp electroencephalography (EEG) is classically seen as an unspecific entity in terms of its association with both epilepsy and distinct aetiologies. This could be due to a lack of standardization in the assessment of slowing morphology, in distinction to precise guidelines utilized in the evaluation of pathological activities, such as interictal epileptiform discharges (IEDs). However, consistent features can be identified, which can be informative about the underlying brain pathology and risk of seizures. In this review, we discuss the different kinds of focal slowing, focusing mainly on the delta range (0.5–4 Hz), their respective pathological processes and their prognostic value in terms of likelihood to be associated with acute seizures or epilepsy. We then present new findings on source localization of focal slowing. Last, we review recent evidence of interaction between focal slow activities and epileptic activities. Our didactic approach will guide readers through important aspects of an EEG entity that might have been neglected despite past and recent observations of its relevance in epilepsy.

## Highlights

• “Rhythmic” and “polymorphic” delta activity reflect focal slowing with stable and varying shape across time, respectively.
• Rhythmic delta activity is more associated with epilepsy than is polymorphic delta activity.
• Recent research suggests mechanistic interplay between focal slowing and epileptic activity.

## 1 Introduction

Electroencephalography (EEG) plays a central role in the diagnosis and management of patients with epilepsy. Within the current operational framework for diagnosing epilepsy (Fisher et al., 2014), EEG is particularly important in the evaluation of a patient with a first episode suspect of an epileptic seizure. Indeed, finding of interictal epileptiform discharges (IEDs) raises the probability of further seizures enough that a diagnosis of epilepsy can be made (Bouma et al., 2016, Baykan et al., 2025). Unfortunately, while IEDs are a specific marker of epilepsy, routine EEG displays epileptogenic abnormality in only 25–43 % of new onset epilepsy cases (De Stefano et al., 2023, King et al., 1998) and, overall, EEG has thus a low sensitivity for epilepsy (Smith, 2005). However, IEDs are not the only abnormality observed in the EEG of patients with epilepsy; pathological focal slowing of EEG activity is at least as common (Schreiner & Pohlmann-Eden, 2003).

During wakefulness, the dominant rhythm of background EEG consists in alpha activity, most predominant in posterior leads. The range of alpha activity in adults – the population of interest in the current review – spans 8 to 13 Hz. Any discernible electrophysiological entity that stands out from this background should be carefully evaluated and eventually defined as a pathological activity, variant or artefact. For instance, IEDs are typical pathological entities seen in people with epilepsy. Studies have identified 6 criteria to define an IED recorded with scalp EEG (Kural et al., 2020a, Kural et al., 2020b, Nascimento and Beniczky, 2023) and there is even evidence that certain of these morphological features are more relevant than others in predicting the diagnostic accuracy (Kural et al., 2021). Hence, there is a relatively high level of standardization in the morphological definition of IEDs.

Conversely, focal slowing exhibits more morphological variability. There is no official definition of focal slowing (Kane et al., 2017), but it usually reflects the occurrence of oscillatory activity within the low-frequency range, either delta (0.5–<4 Hz) or theta (4–<8 Hz) band. Focal slowing is a non-specific finding in terms of underlying aetiology and association with epilepsy. Various brain pathologies such as stroke, encephalitis or mass lesions can exhibit focal slowing, and in turn the presence of focal slowing does not necessarily represent a marker of epileptic activity. Nevertheless, certain features can help distinguish between these aetiological entities, as discussed below. In epilepsy, evidence suggests that focal slowing within the delta frequency band is restricted to the 2–4 Hz range, which is increased in the seizure-onset zone, while the lower band <1 Hz has been shown to be decreased near the seizure-onset zone (Lundstrom et al., 2019). Furthermore, people with epilepsy are more likely to exhibit focal delta slowing than focal theta slowing (Tao et al., 2011). In comparison to focal delta slowing, theta slowing also displays a lower lateralization value (Gambardella et al., 1995). For instance, in 131 patients who benefitted from temporal lobe resection for temporal lobe epilepsy (TLE), 4 exhibited lateralized slowing with theta predominance against 69 with delta predominance (Koutroumanidis et al., 2004). Moreover, theta activity is also more frequently associated with normal variants of uncertain significance (Zumsteg et al., 2004), which can make pathological slowing difficult to differentiate from physiological activity within this frequency band (Fox et al., 2022). Finally, in the hippocampus, theta activity has a physiological role as it tracks memory function (Joensen et al., 2023). For instance, Lega *et al.* found a peak at 2.5–5 Hz and 5.5–10 Hz, which they called ‘low’ and ‘high’ theta respectively, in intracranial recordings of people undergoing an episodic memory task. They observed that low theta is higher during successful memory encoding, in contrast to fast theta, which is not (Lega et al., 2012). For these reasons, this review focuses mainly on delta slowing in focal epilepsy. Unless stated otherwise, studies reported here were based on adult population. Indeed, brain development is associated with changes in brain rhythms (Cellier et al., 2021), which precludes a direct extrapolation of findings from adults to children.

The terms “focal slowing” and “delta activity” can be confusing and are typically used interchangeably in the literature to refer to different electrophysiological entities. In this review, focal slowing encompasses two distinct activities: polymorphic delta activity and rhythmic delta activity. In the following review, we will use this binary terminology when provided in the literature that we cite, or when a specific type can be inferred from the description of the cited article. We will use “focal slowing” when the distinction is unclear. Hence, we first aim to present the established knowledge on polymorphic delta activity, generally reflecting an underlying structural lesion, and no specific relations to epileptic activity, vs rhythmic delta activity (RDA), which has a strong association with epileptic seizures. We then review more recent works on the generative deep sources of focal slowing in focal epilepsy using combined scalp and intracranial recordings. In the last part, we address the most recent advances on the interaction between slow oscillations and epileptic activity (Meisenhelter et al., 2021, Sheybani et al., 2021, Westin et al., 2022), slow waves (SWs) and epileptic activity (Sheybani et al., 2023b, Sheybani et al., 2025a, Sheybani et al., 2025b), and the concept of paroxysmal slow cortical activity (Milikovsky et al., 2019, Zelig et al., 2021).

## 2 Current knowledge on focal slowing in focal epilepsy

First and foremost, it is important to note that the literature usually reports prevalence rates of focal slowing in patients with epilepsy, while the prevalence of epilepsy in EEGs displaying focal slowing is underreported. This publication bias can contribute to a misrepresentation of the true association between epilepsy and focal slowing or wrongly suggest an exclusive association between them. It is thus important to acknowledge that focal slowing in the theta and delta ranges can be seen in up to 44 % and 26 % respectively of a non-epileptic, control population above 60 years old evaluated for syncope (Hughes and Zialcita, 2000, Pirgit and Beniczky, 2024). Hence, focal slowing can also be observed in people without epilepsy.

Focal slowing encompasses polymorphic delta activity and RDA. Research works that meticulously differentiated these two patterns are relatively new (Gaspard et al., 2013), which can make the comparison between past and recent studies difficult. However, clear trends can be derived regarding their association with epilepsy. We discuss these two patterns in more detail in the next sections.

### 2.1 Polymorphic delta activity

**Fig. 1:**
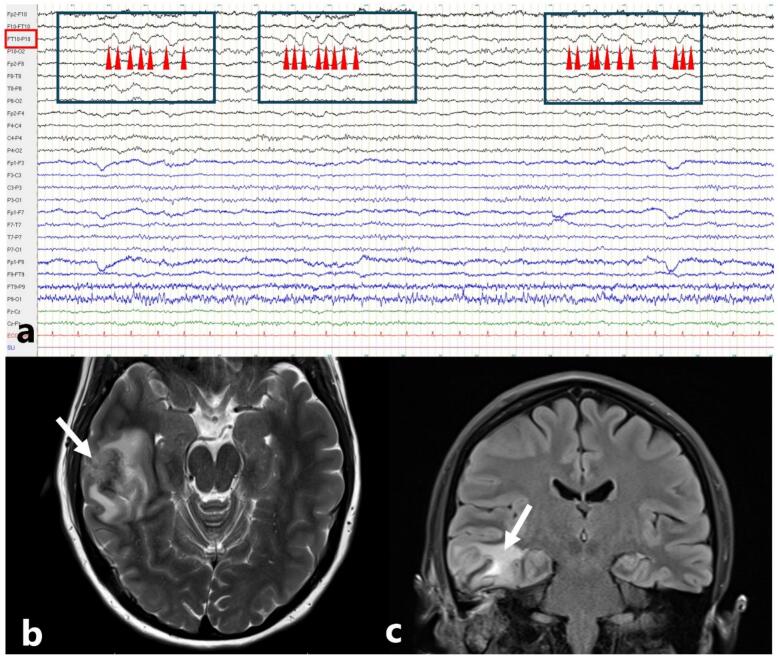
Polymorphic delta activity in a patient with epilepsy consecutive to temporal abscess (a) Scalp EEG displaying 3 instances of right, polymorphic focal slowing centred over temporal leads (FT10-P10). Note the inhomogeneous shape of delta activity across the 3 boxes. Arrowheads point towards zero-crossings; as seen, these are not regular across time, reflecting more heterogenous frequency content than the activity depicted in Fig. 3, Fig. 4. Note also the more variable amplitude of polymorphic delta activity. (b) Axial T2 and coronal FLAIR MRI exhibiting the right temporal abscess.

**Fig. 2:**
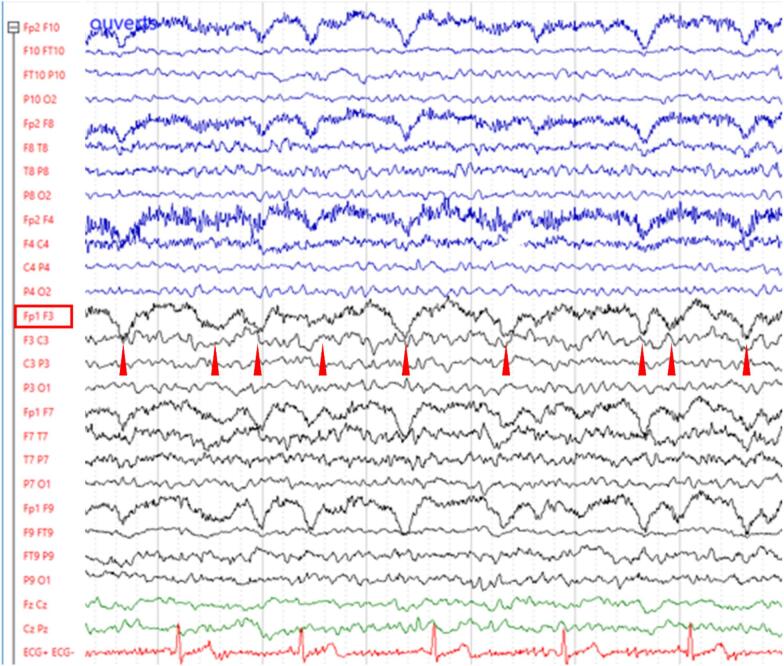
Polymorphic delta activity In this other example of polymorphic delta activity, the variable latency between troughs of the delta oscillation (red arrowheads) reflects the polymorphism, i.e., heterogeneity of the frequency content of delta activity, which contributes to the polymorphic aspect of the oscillation. (For interpretation of the references to colour in this figure legend, the reader is referred to the web version of this article.)

Polymorphic delta activity (Fig. 1, Fig. 2) reflects a slowing within the delta range (0.5-<4 Hz) with variable shape of the oscillation across time. Typically, each period is a loose approximation of a neighbouring cycle, in contrast to the monomorphic aspect of RDA, as discussed below. The incidence of epilepsy in patients with focal, polymorphic delta slowing is relatively low, but not negligible – up to 20 % of critically ill patients who exhibit focal slowing on urgent or continuous EEG present a seizure during hospital stay (Gaspard et al., 2013).

Polymorphic delta activity has been historically associated with an underlying brain lesion (Joynt et al., 1965), especially in the case of cortical disconnection with white matter lesions (Gloor et al., 1977). Quantitative analyses using imaging studies confirmed the association. Indeed, in an early, prospective study of 100 patients with polymorphic delta activity who benefitted from a CT scan within 2 weeks after the EEG, only a third had normal or non-focal abnormalities on imaging studies (Marshall, 1988). A subsequent study on children further showed that 50 % display imaging abnormalities (42 CT scans, 21 MRI, 17 both imaging modalities) concordant with polymorphic delta activity (Maytal et al., 1993). The association was weaker in a more recent study on over 500 adult patients, among whom only 37 % with a focal lesion (imaging modality not disclosed) also had a focal slowing on EEG, presumably of polymorphic delta activity type (Lemoine et al., 2023). The association with a brain structural pathology is also true in epilepsy. In a large cohort of 253 children with non-syndromic epilepsy (Noh et al., 2013), 40 % of children overall exhibited MRI abnormality concordant with the location of polymorphic delta activity. Interestingly, in children without IEDs, the side of MRI abnormality corresponded to that of the polymorphic delta activity in 70 %, reinforcing the concept that slowing reflects an underlying brain lesion. Of note, 54 % of patients presented with focal slowing (with or without IEDs).

Whether focal slowing predicts seizure recurrence after epilepsy surgery deserves also to be mentioned. After surgery, delta power at rest is higher in patients with recurring seizures than that in seizure-free patients or healthy controls (Schönherr et al., 2017). Hence, this could suggest a role of delta activity in predicting seizure recurrence after surgery. The morphological appearance, i.e., whether polymorphic or rhythmic, needs to be clarified. Furthermore, confounders could also contribute to this result. For example, since delta activity can persist several days after a seizure (Boly et al., 2017), it would be important to verify whether delta activity is not simply reflecting the recent occurrence of seizures, rather than predicting their recurrence. The same caveat should be raised in other studies in general.

The presence of IEDs on EEG following a first seizure is predictive of seizure recurrence (Krumholz et al., 2015, Baykan et al., 2025) – is it also the case for polymorphic delta activity? Surprisingly, the performance of focal slowing in predicting seizure recurrence after a first seizure has not been thoroughly investigated, as this feature is usually part of the definition of “abnormal EEG”, together with IEDs, in studies that investigated such biomarkers (Bonnett et al., 2022, Debicki, 2017). A study looked at the predictive value of Paroxysmal Slow Wave Events (PSWE), defined as episodes of ≥ 5 s with a median frequency content < 6 Hz (Zelig et al., 2021). The incidence of PSWE was significantly higher in patients with seizure recurrence than in those without recurrence or healthy controls. EEGs performed better when obtained earlier (within 72 h of the initial seizure) – similarly to better prognostic value of early EEG in identifying IEDs (King et al., 1998, Krumholz et al., 2015) – yet formal statistical testing will be necessary to confirm this observation. To note, the possibility that a brain lesion could be a confounding factor that predicts both the risk of seizure recurrence (Krumholz et al., 2015) and PSWE should also be methodically tested. Related to this point, the incidence of PSWE was higher in patients treated with antiseizure medication (ASM) who presented seizure recurrence, compared to those who remained seizure-free (also treated with ASM) (Zelig et al., 2021). Since ASM was introduced following either brain imaging or EEG, this analysis thus mitigated the risk that PSWE is a mere biomarker of a brain epileptogenic lesion. The same study found that patients with recurring seizures had significantly higher delta (1–4 Hz) power (as well as lower beta [12–20 Hz] and low gamma [20–30 Hz] band) than those without recurrence, but whether this holds a predictive value remains to be tested (Zelig et al., 2021). In another study, there was a non-significant trend for seizure recurrence in patients with unilateral delta or theta activity (Schreiner & Pohlmann-Eden, 2003). Hence, polymorphic delta activity might be related to epilepsy, but further studies are necessary to clarify its predictive value after a first seizure.

### 2.2 Rhythmic delta activity

**Fig. 3:**
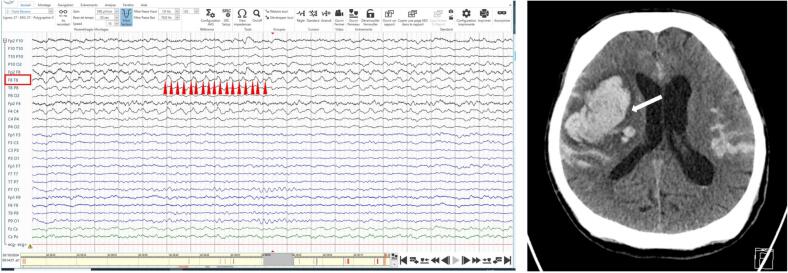
Lateralized rhythmic delta activity in a patient with right acute temporal haemorrhage *Left* Lateralized rhythmic delta activity with sharp morphology (RDA + *S*) can be seen on right temporal derivates (F8-T8 and F4-C4). Note the regular shape of the oscillation, in contrast to that of Fig. 1. Arrowheads point toward zero-crossings, reflecting the more homogenous frequency content of rhythmic delta activity. *Right* Intracerebral right hemispheric haemorrhage (white arrow). This patient presented several seizures – of presumably symptomatic origin consecutive to the haemorrhage – and the EEG displayed an initial improvement after introduction of antiseizure medication.

In contrast to polymorphic delta activity, rhythmic delta activity (RDA, Fig. 3) corresponds to a regular oscillation with monomorphic appearance. Intermittent RDA is subdivided into intermittent generalized RDA (GRDA), which encompasses the previous entities of frontal and occipital intermittent RDA (FIRDA and OIRDA), respectively (Tao et al., 2020), and lateralized RDA (LRDA). The formulation of LRDA, which is part of the rhythmic and periodic patterns (RPPs) included in the American Clinical Neurophysiology Society’s standardized critical care EEG Terminology (Hirsch et al., 2021), has been studied most intensively in critically ill patients (Gaspard et al., 2013), but the pattern was initially used in non-critically ill patients under the previous name of Temporal Intermittent RDA (TIRDA) (Tao et al., 2020). Hence, although LRDA encompasses now TIRDA and both are used interchangeably in the literature (Lemus et al., 2024), there are slight differences between these two patterns: LRDA encompasses all hemispheric monomorphic rhythmic delta activities, while TIRDA is limited to the temporal region; LRDA is most commonly seen in patients with ischaemic and haemorrhagic stroke or critically ill patients in general, while TIRDA is classically used in out-patients or not-critically ill population and displays a high specificity for epilepsy (Reiher et al., 1989). For these reasons, we discuss TIRDA and LRDA separately.

#### 2.2.1 Temporal intermittent rhythmic delta activity (TIRDA)

TIRDA consists in short trains of RDA that usually occurs during drowsiness or sleep in anterior temporal leads (Kane et al., 2017). In contrast to polymorphic delta activity, it is associated with TLE (Brigo, 2011, Geyer et al., 1999, Kane et al., 2017). Indeed, the specificity of TIRDA for epilepsy with focal seizures with impaired awareness and anterior temporal IEDs was documented as high as 98.7 % in a large cohort of 127 patients and 115 controls (i.e., neurological patients without focal seizures with impaired awareness) (Reiher et al., 1989). This was further confirmed in a subsequent survey that analysed 12,198 EEGs and found TIRDA in 0.3 % of them (n = 33, 27 patients); focal seizures with impaired awareness were diagnosed in 23/27 patients with TIRDA (Normand et al., 1995). Ensuing studies further supported the association with TLE by including patients with extra temporal lobe epilepsy (ETLE). In a large cohort of 129 patients, TIRDA was significantly associated with TLE; 86 % of medial TLE (MTLE) exhibited the pattern, while it was not seen in patients with strictly lateral TLE or ETLE (Di Gennaro et al., 2003). Since then, a case of extra-temporal TIRDA has been described (Freund et al., 2022), indicating that the presumably exclusive association between MTLE and TIRDA need to be confirmed by intracranial recordings.

TIRDA also exhibits a good lateralization value. Across 56 patients with TLE and medial atrophy, 72 % patients displayed lateralized TIRDA against only 9 % bilateral TIRDA (Gambardella et al., 1995). Similarly, a low but significant correlation between positron emission tomography (PET) hypometabolism and TIRDA has been identified in people with intractable TLE (Altay et al., 2005). Altogether, TIRDA has thus a good predictive value for TLE, in particular of medial origin, and is a relatively good lateralization entity.

#### 2.2.2 Lateralized rhythmic delta activity (LRDA)

As previously mentioned, LRDA is a pattern that is conceptually linked and mostly – if not exclusively – studied in critically ill patients (Fong et al., 2023, Gaspard et al., 2013, Gupta et al., 2025, Husari et al., 2021, Rodriguez Ruiz et al., 2017).

LRDA is found in a significant proportion (up to 5 %) of critically ill patients (Gaspard et al., 2013). It is associated with an imaging abnormality in 87 % of cases and with a concordant MRI finding in 66 % (Alzawahmah et al., 2022). Although the prevalence of epilepsy in those who present this pattern is high – up to 63 % of critically ill patients who undergo urgent or continuous EEG and exhibit this pattern will also experience an epileptic seizure during their stay (Gaspard et al., 2013) – its incomplete association with seizure indicates that the introduction of ASM should be decided on an individual basis and reviewed according to the electro-clinical evolution. The earliness of LRDA emergence in the course of an acute disease is a prognostic factor. Indeed, the appearance of RPP – of which LRDA is an entity – occurs earlier in critically ill patients after cardiac arrest who eventually present a poor outcome (severe disability, coma or death) than in those with good outcome (Van Putten et al., 2024). These results indicate that LRDA should not consistently be assumed to reflect the presence of seizures but could rather be a biomarker of a particularly severe brain condition.

Hence, in patients who undergo urgent or continuous EEG monitoring, 63 % of those with LRDA will present an epileptic seizure during their stay, and this is much higher than the 20 % of those with polymorphic delta or theta activity (Gaspard et al., 2013). In another large survey on 12,450 critically ill patients undergoing continuous EEG monitoring, it was shown that, while LRDA is significantly associated with seizures, it is the only RPP not associated with status epilepticus (Snider et al., 2023).

Certain features of LRDA make it particularly predictive of seizures. These features are called “modifiers” and include, among others, the frequency or a sharp contour of RDA (see Fig. 3 for an example of RDA + sharp, abbreviated S) (Hirsch et al., 2021). Indeed, in a large cohort of 4,772 critically ill patients, the presence of LRDA was associated with seizures, but only with frequencies of 1.5 Hz or greater, or when associated with specific modifiers (Rodriguez Ruiz et al., 2017). Altogether, there is thus strong evidence supporting that certain characteristics of LRDA, including frequency and presence of specific modifiers, should be considered a probable marker of an ongoing seizure in the “ictal-interictal continuum” (Johnson & Kaplan, 2017). Interestingly, and in keeping with this association between LRDA and epileptic activity, intracranial IEDs are associated with an increased delta power on scalp EEG in particular at a frequency ≥ 1.4 Hz (De Stefano et al., 2022).

#### 2.2.3 FIRDA and OIRDA

The pattern previously called FIRDA is a rather non-specific finding both in regard to the aetiology of the underlying disease and its topographical specificity (Brigo, 2011). In a large cohort of 7,689 EEGs performed at a university hospital, FIRDA was found in 1 % of patients, and about half of them did not have any significant imaging abnormality (Kim et al., 2021). Of those without a structural brain lesion, the confirmed diagnosis was metabolic encephalopathy (54 %), neurodegenerative disease (16 %), hypoxic encephalopathy (14 %), systemic infection without CNS involvement (11 %), and encephalitis (5 %), illustrating the heterogenous causes of FIRDA. Unilateral FIRDA, in contrast to bilateral FIRDA, is more closely associated with seizures and epileptiform abnormalities, although a formal cut-off between bilateral and unilateral FIRDA remains to be defined (Cerrahoğlu Şirin et al., 2019, Gaspard et al., 2013, Mina et al., 2019).

In individual cases, FIRDA can reflect the occurrence of epileptiform discharges from deep sources, such as the medial temporal lobe (Getter et al., 2023). In this case report, asymmetrical FIRDA was recorded concurrent with temporal discharges and disappeared after radio-frequency ablation of the hippocampal focus. Similarly, in a series of 94 in- and outpatients who benefitted from a scalp EEG for various reasons and exhibited FIRDA, 39 had epilepsy (Mina et al., 2019). While only 37 % of them had a symmetric FIRDA, a symmetric pattern was found in 60 % of non-epileptic patients (Mina et al., 2019). Thus, although this remains a hypothesis given the lack of data, there is suggestive evidence that lateralized FIRDA could be the frontal equivalent to TIRDA, although the association between TIRDA and epilepsy is much clearer, as previously discussed.

OIRDA is generally seen in children and has been found more frequently in children with epilepsy, despite a relatively small effect size. Indeed, a study on young subjects (1–22 year-old) reported a rate of people with any type of seizure of 1.7 in people with OIRDA against 1 in people without OIRDA (Gullapalli & Fountain, 2003). Although OIRDA was thought to be closely associated with generalized epilepsies (Gullapalli & Fountain, 2003), rather than focal epilepsies as is the case for LRDA, more recent evidence has indicated that it can also be seen in children with focal epilepsy (LaBarbera and Nie, 2021, Watemberg et al., 2007). Across 697 EEGs, OIRDA was found in 24 recordings (3 %) and half of them exhibited epileptiform abnormalities; these were focal in all but one study (Watemberg et al., 2007). Among children with childhood absence epilepsy, OIRDA is found in 21 % (Dlugos et al., 2013) and represents the only non-physiological focal slowing activity that does not put the diagnostic into question. On relatively small series that included children with generalized and focal epilepsy, OIRDA can be unilateral in up to 21 % of cases (Gullapalli & Fountain, 2003).

## 3 Brain generators of focal slowing

**Fig. 4:**
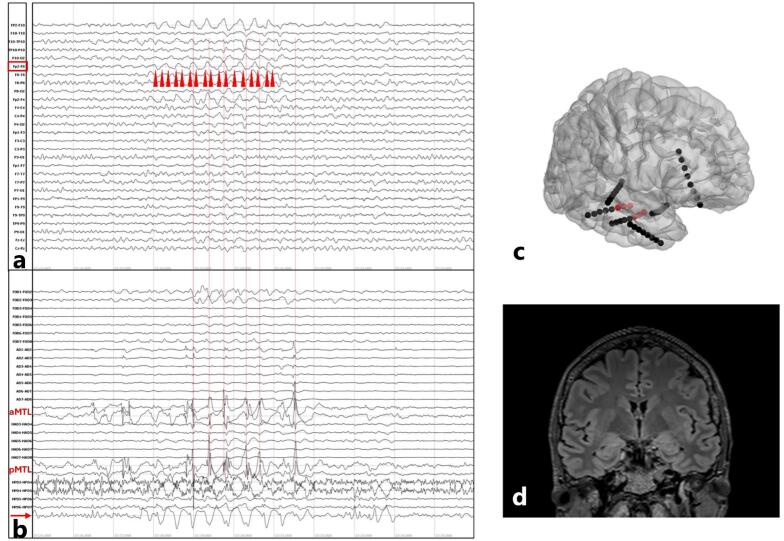
Combined scalp-intracranial EEG recording shows the deep source of surface-recorded lateralized rhythmic delta activity (a) The centre of the page shows 3 s of lateralized rhythmic delta activity centred over right frontal-temporal area (Fp2-F10, Fp2-F8, Fp2-F4). The first 5 vertical bars align with deep-recorded epileptiform discharges on (b), while the 6th is an instance where deep epileptiform discharge is not reflected into a paroxysmal event on the scalp EEG. For a and b, arrowheads point towards zero-crossings, reflecting the more homogenous frequency content of rhythmic delta activity. aMTL = anterior mesial temporal lobe. pMTL = posterior mesial temporal lobe. aMTL and pMTL traces correspond to electrodes depicted in red in panel c (bipolar montage). The signal indicated by an arrow corresponds to a posterior neocortical temporal electrode.(c) Intracranial montage. The hippocampal electrodes with maximal activity during the epileptiform discharges are depicted in red. (d) Coronal FLAIR MRI exhibiting asymmetry of size of the right hippocampus, in contrast with the left hippocampus, compatible with an epileptogenic lesion. Adapted from (De Stefano et al., 2020).

Pathological activity, mainly within the delta range, but also theta range (Gaspard et al., 2013), is classically associated with subcortical white matter or brainstem lesion and slower “disconnected” cortical activity (Gloor et al., 1977, Kaplan and Rossetti, 2011, Sheybani et al., 2012). More recent evidence based on combined scalp and intracranial recordings has provided insight into the grey matter source of LRDA. In a patient with TLE, combined recording revealed that scalp-recorded LRDA was synchronized with RDA in the lateral temporal neocortex, while the medial temporal leads did not show such activity (Serafini et al., 2016). In line with this, some evidence suggests that abundant LRDA (including polymorphic slowing in that study) is more frequently associated with neocortical TLE, while uncommon or occasional LRDA is rather associated with MTLE, although formal quantification is needed to clarify the association (Tao et al., 2011). Another report on 3 patients suggested that the area of the brain parenchyma that records an IED, estimated by the number of intracranial contacts where it is visible, and the amplitude of the IED determine whether the intracranial graphoelement is captured by scalp EEG, usually in the form of a spike-and-wave complex (Sakata et al., 2022). Interestingly, in one patient, IEDs in lateral temporal contacts were captured by scalp EEG, while a simultaneous seizure originating in the medial temporal lobe was not (Sakata et al., 2022). Nevertheless, the association between lateral temporal neocortical discharges and LRDA is not exclusive, as shown by another report of a single patient with MTLE, in whom combined high-density scalp EEG and intracranial recording showed a hippocampal source of the scalp-recorded LRDA (Fig. 4) (De Stefano et al., 2020). Similarly, one study reported increase in scalp-recorded delta power ≥1.4 Hz associated with intrahippocampal recording of IEDs (De Stefano et al., 2022).

Focal slowing could even contribute to the identification of the epileptogenic zone. In a cohort of 15 children who benefitted from successful surgical outcome (Engel I) for drug-resistant epilepsy of heterogenous lobar origin, electrical source imaging of rhythmic and arrhythmic focal delta or theta activity was concordant with the resected area in 60 %, i.e., within 5 mm of resection margins (Baldini et al., 2020), again reinforcing the spatial concordance between this pattern and epileptic activity. Hence, rhythmic focal activity within the delta-theta range, as recorded on scalp EEG, could be the surface equivalent of intracranial IEDs or ictal discharges in the lateral or medial temporal lobe.

An alternative hypothesis could be related to changes of cortical activity during hippocampal seizures. In a rodent MTLE model, neocortical neurons enter a bistable state made of an UP- and DOWN-states during hippocampal seizures, and this bimodal activity is associated with a slow oscillation on cortical recordings (Motelow et al., 2015, Sieu et al., 2024). Thus, regions that are functionally connected to the seizure-generating area might contribute to the focal slowing observed during seizures.

## 4 Recent advances on the interaction between slow activity and epileptic activity

So far, we have discussed epidemiological evidence of the association between polymorphic delta activity, RDA and epileptic activity. In the following section, we discuss the relationship between epileptic entities and SWs, whether they are sampled during wakefulness or sleep. These SWs presumably share core generative mechanisms with polymorphic delta activity and RDA. These mechanisms rely on the neuronal membrane bistability during SWs, leading neurons to oscillate between an active state, so called UP-state, and an inactive state, so called DOWN-state (Cash et al., 2009, Massimini et al., 2024, Sanchez-Vives et al., 2017). Further studies are needed in the field of epilepsy, as this has been mainly investigated in the field of stroke (Massimini et al., 2024, Sarasso et al., 2020). This does not imply that physiological and pathological SWs are triggered or induced by the same mechanisms, only that they reflect the same neuronal activity, i.e., bistable membrane potential.

**Fig. 5:**
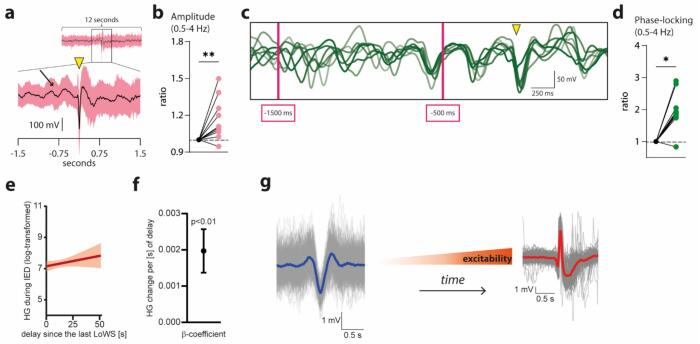
Interaction between slow waves and interictal epileptiform discharges (a) Average intracranial EEG activity in one patient, one electrode, locked to the peak of detected interictal epileptiform discharges (IEDs, peak at yellow triangle). The black arrow indicates an oscillation (0.5–4 Hz, delta band) that anticipates the IED. (b) Across participants, there was a significant increase in slow oscillation power before IEDs. (c) Seven representative trials showing the activity filtered between 0.5–4 Hz before an IED (indicated by the yellow triangle). At −1500 ms, all trials show a different phase (they are not aligned), while at −500 ms, all trials show a similar phase (they are superimposed). This indicates that IEDs occur at a fixed phase of underlying oscillation, across repetitions of several IEDs. This systematic occurrence of an entity (here, IEDs) at a particular phase of another entity (i.e., slow oscillation) is called “phase-locking”. (d) Across participants, there was a significant increase in phase-locking before IEDs. (e) Representative example of a correlation between delay since the last local wake slow waves (LoWS, x-axis) and excitability during IEDs, measured by high-gamma (HG) power. (f) Across participants, this correlation was significant. (g) The previous result indicates that the longer the latency since the last LoWS, the higher the excitability during the following IED, as illustrated by the progressively larger triangle between LoWS (blue) and IED (red). Panels a-d: adapted from (Sheybani et al., 2021). Panels e-g: adapted from (Sheybani et al., 2023).

Recent data suggest possible mechanistic links between focal slow activity and interictal discharges. Evidence has highlighted that IEDs occur during a background of slow oscillatory activity (delta and theta ranges), including in experimental models of epilepsy (Frauscher et al., 2015, Meisenhelter et al., 2021, Sheybani et al., 2019, Sheybani et al., 2021, Westin et al., 2022). In line with this, the epileptogenic hemisphere exhibits more slow wave activity during sleep (Sheybani et al., 2023a). Interestingly, not only do IEDs occur during such vulnerable windows (Fig. 5a-b), but they are precisely phase-locked to the slow oscillation background (Fig. 5c-d) (Sheybani et al., 2021). In the latter study, it was further shown that high-gamma power, an intracranial EEG proxy for local neuronal activity (Ray et al., 2008), shows increased phase-amplitude coupling to delta oscillations before IEDs. This aligns with the hypothesis that slow oscillations could coordinate neuronal activity in such a manner that increased synchronized neuronal discharges during UP-states favour IEDs (Frauscher et al., 2015). The observation that IEDs are more likely during non-rapid eye movement sleep stage 3 (NREM 3) (Ng & Pavlova, 2013), i.e., when slow wave activity is higher than in any other sleep or wake states, is in line with this hypothesis and, altogether, suggests that slow oscillations contribute to IED generation. To note, this would hold if we can assume that pathological low frequency (delta-theta) activity is similar to sleep slow waves. As already said, in the field of stroke, some evidence indeed supports that focal, low frequency activity reflects intrusion of sleep-related entities during wake periods (Massimini et al., 2024). The existence of alternating enhanced (UP-) and decreased (DOWN-state) neural activity, both in the field of stroke (Sarasso et al., 2020) and epilepsy (Sheybani et al., 2023b) argues in favour of this hypothesis.

However, not all results are concordant. For example, slow wave activity fluctuates during NREM 3 and the period of NREM 3 that is characterized by the highest level of slow waves is actually the one with the lowest rate of IEDs (Zubler et al., 2017). This contrasts with results from Frauscher et al. (2015), where SWs of high amplitude were associated with more IEDs. However, the discrepancy could be due to the fact that incidence of overall IEDs (i.e., occurring during background, incl. those in the temporal vicinity of SWs, as in Zubler *et al.*) is a poor proxy of those occurring only locked to SWs (as in Frauscher *et al.*). It could also be due to the different populations being studied: in Zubler *et al.*, only patients with type 2 focal cortical dysplasia were included. In the second case, this study (Zubler et al., 2017) suggests that sleep slow waves might not facilitate IEDs, at least in some types of epilepsy. Similarly, more recent evidence suggests that slow waves during wakefulness offset excitability associated with IEDs (Sheybani et al., 2023b). Indeed, people with drug-resistant focal epilepsy exhibit slow waves typical of sleep during wakefulness. In this population, it was shown that the longer the delay since the last wave, the higher the excitability associated with IEDs (Fig. 5e-g), as if any restorative function of slow waves dissipates with time (Sheybani et al., 2023b). Further studies are thus necessary to disentangle the relationship between slow waves, epileptic activity and network excitability (Sheybani et al., 2025a). Of note, the incidence of these wake slow waves in medial temporal structures was no different in patients with and without MTLE, the latter group including lateral temporal lobe epilepsy (LTLE) and ETLE (Sheybani et al., 2023b). This is important, since evidence suggests that the hippocampus generates more delta activity than other brain areas (Frauscher et al., 2018). Hence, any region-wise correlation between slow oscillatory activity and IEDs should be carefully interpreted. Last, a recent study provided evidence that the interaction between IEDs and slow waves could have a prognostic value in terms of post-surgical outcome in people with drug-resistant focal epilepsy (Sheybani et al., 2025b). Short temporal delays between IEDs and subsequent slow waves were found in epileptogenic zones, further reinforcing the potential role of slow waves in controlling excitability in epilepsy (Sheybani et al., 2025a).

Slow oscillatory activity has also been observed, and artificially triggered, in people with brain lesions. In these patients it is proposed that the slow activity disrupts rehabilitation after stroke (Massimini et al., 2024, Rosanova et al., 2018, Sarasso et al., 2020, Sarasso et al., 2025). This appearance of focal slowing in or nearby brain injuries has been hypothesized to reflect neuronal bistability – a state in which neurons oscillate between periods of depolarization (i.e., excitable) and hyperpolarization (i.e., inhibited) – similar to that during sleep slow waves (Massimini et al., 2024). The emergence of neuronal bistability is suggested to be consecutive to lesion to the ascending activating fibers, cortico-cortical tracks, or both (Massimini et al., 2024). This again underlines the role of white matter lesion in the expression of focal slowing (Gloor et al., 1977). To note, the hypothesis that slow activity following brain lesion disrupts rehabilitation contrasts with the improvement of fine motor skills upon optogenetically-induced slow waves during sleep in a rodent model of stroke (Facchin et al., 2020). Nevertheless, the fact that, in the latter, slow waves were evoked during sleep – when they are expected to occur – might explain their beneficial function.

Another type of focal slowing, which is still being experimentally investigated, is PSWE. As indicated before, this corresponds to episodes of ≥5 s during which the median power frequency is <6 Hz. PSWEs are associated with both Alzheimer disease and epilepsy (Milikovsky et al., 2019), but not with Parkinson’s disease (Milikovsky et al., 2023). Their incidence was shown to be higher in patients who had further seizures after a first event (Zelig et al., 2021). Further work should clarify the differences between PSWEs and the more classical LRDA or polymorphic delta slowing, both in terms of their morphology and their relationship with epilepsy.

## 5 Conclusion and perspectives

Slow oscillatory activities are utilized both in the context of outpatient monitoring and diagnosis and inpatient prognosis, especially in epilepsy monitoring and critical care units. Although they encompass a more heterogeneous group than IEDs, specific characteristics can help cluster them into distinct entities. In particular, the polymorphic vs. regular aspect is a key feature to assist the interpretation of this pattern.

Polymorphic delta activity is generally associated with an underlying structural abnormality but is not specific to epilepsy. Conversely, TIRDA in out-patients generally reflects the presence of TLE, in particular of medial origin, while LRDA – its equivalent in critically ill patients – displays a strong, but not exclusive, association with seizures. Whether these patterns – TIRDA and LRDA – have the same generative mechanisms in both situations – non-critically ill and critically ill patients respectively – will need to be clarified in future research.

The fact that IEDs are most likely to occur during NREM 3, when sleep SWs are most frequent, has led to the hypothesis that slow waves could facilitate the expression of IEDs. However, during NREM 3, when IEDs are more likely to occur, SWs occurrence fluctuates and the period characterized by high content in SWs is marked by the lowest IED rate, arguing against an immediate facilitation of IEDs by sleep SWs. Similarly, a protective function of slow waves during wakefulness has been recently suggested, based on intracranial recording in people with focal epilepsy. Hence, the relationship between slow waves and IEDs remain uncertain in the face of studies with divergent observations. Further research is thus warranted.

Other areas of research that deserve further work include the value of focal slowing in predicting the recurrence of seizures. A certain pattern of focal slowing (PSWE) supports such a potential predictive performance.

## Declaration of competing interest

The authors declare the following financial interests/personal relationships which may be considered as potential competing interests: MS and SV have shares in Epilog. LS, PDS and PM report no conflict of interest.
